# Supplementary material for: Dimensional reduction and incommensurate dynamic correlations in the S = ½ triangular-lattice antiferromagnet Ca3ReO5Cl2
Source: Nat Commun. 2022 Oct 23;13:6310. doi: 10.1038/s41467-022-33992-5 (PMC9588769; doi:10.1038/s41467-022-33992-5)
Supplement: Supplementary file 1 — Supplementary Information [file 41467_2022_33992_MOESM1_ESM.pdf]

Dimensional reduction and incommensurate dynamic correlations in the $S = \frac{1}{2}$ triangular-lattice antiferromagnet $Ca_3ReO_5Cl_2$ (Supplementary Information)

S. A. Zvyagin,[1,*] A. N. Ponomaryov,[1,†] J. Wosnitza,[1,2] D. Hirai,[3] Z. Hiroi,[3] M. Gen,[3] Y. Kohama,[3] A. Matsuo,[3] Y. H. Matsuda,[3] and K. Kindo[3]

[1]*Dresden High Magnetic Field Laboratory (HLD-EMFL) and Würzburg-Dresden Cluster of Excellence ct.qmat, Helmholtz-Zentrum Dresden-Rossendorf, 01328 Dresden, Germany*
[2]*Institut für Festkörper- und Materialphysik, TU Dresden, 01062 Dresden, Germany*
[3]*Institute for Solid State Physics, University of Tokyo, Kashiwa, Chiba 277-8581, Japan*

For high-field electron-spin resonance (ESR) experiments single crystals with typical sizes of ca 4x4x1 mm^3 (along the a, b, and c axis, respectively) were used. In Supplementary Figure 1 we show the angular dependence of ESR absorptions for magnetic field applied in the bc plane at a frequency of 370 GHz. The measurement were performed at a temperature of 2 K, clear indicating a large anisotropy of ESR absorption, in particular in the vicinity of the b axis. As revealed, even a small (a few degree) tilting of magnetic field from the b axis results in a significant ESR line splitting. Based on that, we estimate the accuracy of sample orientation in our experiments (Fig. 2 - 4) to be better than ± 5 degree. The anisotropy of the observed ESR absorption in the vicinity of the c axis is not as pronounced. The mode C1 was observed with magnetic field of about 2 T applied along the c direction, while much higher field is needed for the observation of the mode C2$'$ at this frequency.

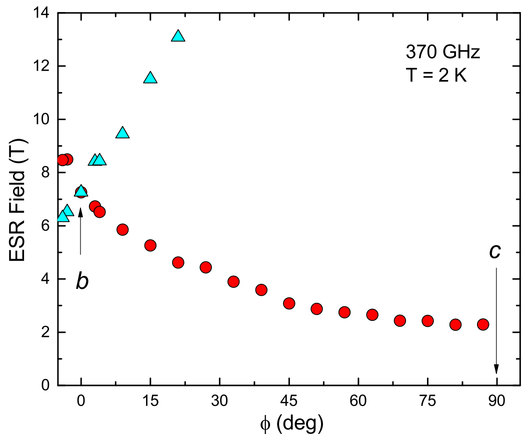

Supplementary Figure 1. ESR angular dependence for magnetic field in the bc plane at a frequency of 370 GHz ($T = 2$ K). Two observed modes are denoted by different colors.

* Corresponding author: s.zvyagin@hzdr.de
† Present Address: Institute of Radiation Physics, Helmholtz-Zentrum Dresden-Rossendorf, 01328 Dresden, Germany.
